# Supplementary material for: Comparison of Masimo Rad-67 SpHb non-invasive hemoglobin monitoring device with complete blood count measurement for use in pregnancy: An observational multi-site cohort study
Source: medRxiv. 2025 Dec 16:2025.12.14.25342241. Preprint. [Version 1] doi: 10.64898/2025.12.14.25342241 (PMC12723978; doi:10.64898/2025.12.14.25342241)

medRxiv preprint doi: https://doi.org/10.64898/2025.12.14.25342241; this version posted December 16, 2025. The copyright holder for this preprint (which was not certified by peer review) is the author/funder, who has granted medRxiv a license to display the preprint in perpetuity.

It is made available under a CC-BY 4.0 International license .

SUPPLEMENTARY MATERIAL

Table S1. Total number of SpHb and CBC measurements at each visit at each site among women with a visit

Site	Timepoint	SpHb (%)	CBC (%)	SpHb and CBC (%)
Pakistan	ANC<20	897 (99.7)	900 (100.0)	897 (99.7)
	ANC20	768 (95.4)	769 (95.5)	756 (93.9)
	ANC28	762 (87.7)	747 (86.0)	723 (83.2)
	ANC36	616 (82.4)	627 (83.8)	568 (75.9)
	PNC6	753 (88.0)	547 (63.9)	529 (61.8)
Kenya	ANC<20	881 (97.9)	899 (99.9)	881 (97.9)
	ANC20	605 (81.1)	464 (62.2)	365 (48.9)
	ANC28	608 (68.1)	528 (59.1)	319 (35.7)
	ANC36	459 (52.1)	491 (55.7)	193 (21.9)
	PNC6	291 (34.7)	493 (58.8)	74 (8.8)
Zambia	ANC<20	873 (97.0)	896 (99.6)	869 (96.6)
	ANC20	657 (88.8)	658 (88.9)	654 (88.4)
	ANC28	788 (97.8)	788 (97.8)	782 (97.0)
	ANC36	684 (99.3)	674 (97.8)	669 (97.1)
	PNC6	672 (83.8)	677 (84.4)	661 (82.4)

ANC: Antenatal care (<20 weeks, 20 weeks, 28 weeks, 36 weeks gestation)
PNC: Postnatal care (6 weeks postpartum)

medRxiv preprint doi: https://doi.org/10.64898/2025.12.14.25342241; this version posted December 16, 2025. The copyright holder for this preprint (which was not certified by peer review) is the author/funder, who has granted medRxiv a license to display the preprint in perpetuity.

It is made available under a CC-BY 4.0 International license .

Table S2. Agreement using Cohen's Kappa and McNemar's test between SpHb and CBC on a binary scale for abnormal hemoglobin levels

**Criteria**	**Cohen's Kappa (p-value)**	**McNemar's Test (χ^2) (p-value)**
Hb<11g/dL or Hb>13g/dL	-0.20 (p=0)	433.18 (<0.001)
Hb<11g/dL or Hb>15g/dL	0.01 (p<0.001)	4460.00 (p<0.001)
Hb<10g/dL or Hb>13g/dL	-0.12 (p<0.001)	519.34 (p<0.001)
Hb<10g/dL or Hb>15g/dL	0.00 (p=0.001)	1457 (p<0.001)
Hb<10g/dl	0.00 (p=0.06)	1245 (p<0.001)
Hb <11g/dl	0.01 (p<0.001)	3445 (p<0.001)
Hb>13g/dl	0.08 (p=0)	1796 (p<0.001)
Hb >15g/dl	Not applicable	Not applicable

medRxiv preprint doi: https://doi.org/10.64898/2025.12.14.25342241; this version posted December 16, 2025. The copyright holder for this preprint (which was not certified by peer review) is the author/funder, who has granted medRxiv a license to display the preprint in perpetuity.

It is made available under a CC-BY 4.0 International license .

Figure S1. Participant eligibility screening and final sample selection

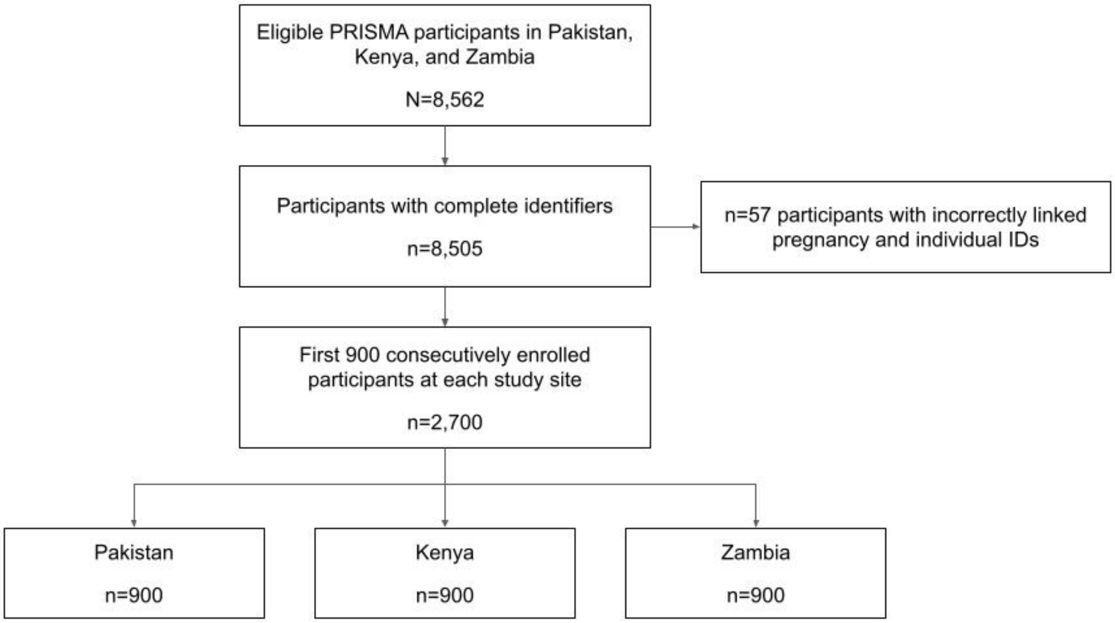

Supplement: 1 [file NIHPP2025.12.14.25342241V1-supplement-1.pdf]
